# Genome-Wide Survey and Expression Analysis of Calcium-Dependent Protein Kinase in *Gossypium raimondii*

**DOI:** 10.1371/journal.pone.0098189

**Published:** 2014-06-02

**Authors:** Wei Liu, Wei Li, Qiuling He, Muhammad Khan Daud, Jinhong Chen, Shuijin Zhu

**Affiliations:** 1 Department of Agronomy, Zhejiang University, Hangzhou, Zhejiang, China; 2 Department of Biotechnology and Genetic Engineering, Kohat University of Science and Technology, Kohat, Pakistan; East Carolina University, United States of America

## Abstract

Calcium-dependent protein kinases (CDPKs) are one of the largest protein kinases in plants and participate in different physiological processes through regulating downstream components of calcium signaling pathways. In this study, 41 CDPK genes, from *GrCPK1* to *GrCPK41*, were identified in the genome of the diploid cotton, *Gossypium raimondii*. The phylogenetic analysis indicated that all these genes were divided into four subgroups and members within the same subgroup shared conserved exon-intron structures. The expansion of *GrCPKs* family in *G. raimondii* was due to the segmental duplication events, and the analysis of Ka/Ks ratios implied that the duplicated *GrCPKs* had mainly undergone strong purifying selection pressure with limited functional divergence. The cold-responsive elements in promoter regions were detected in the majority of *GrCPKs*. The expression analysis of 11 selected genes showed that *GrCPKs* exhibited tissue-specific expression patterns and the expression of *GrCPKs* were induced or repressed by cold treatment. These observations would lay an important foundation for functional and evolutionary analysis of CDPK gene family in *Gossypium* species.

## Introduction

The plant growth and crop production are adversely affected by common stress conditions, such as drought, low temperature and high salinity [1]. Adaptation of plants to these environmental stresses includes the perception of stress signals and subsequent signal transduction, leading to the activation of various physiological and metabolic responses [2], [3], [4]. As a ubiquitous second messenger in cells, calcium (Ca^2+^) plays an important role in the signal transduction pathways. The transient changes of cytoplasmic Ca^2+^ concentration in response to various stresses were sensed and decoded by several Ca^2+^ sensors or Ca^2+^ binding proteins which relayed the signals into downstream response processes such as regulation of gene expression and phosphorylation cascades [5], [6].

There are four major classes of Ca^2+^ binding proteins characterized in plants, including calmodulins (CaM), calmodulin-like proteins (CaML), calcineurin B-like proteins (CBL) and calcium-dependent protein kinases (CDPK) [7], [8], [9]. Among them, CDPKs are the best characterized and are of particular interest, which constitute a large multigene family and are reported to be found throughout the plant kingdom from algae to angiosperms [10]. The CDPK protein possesses four characterized domains, a variable N-terminal domain, a catalytic Ser/Thr protein kinase domain, an autoinhibitory region, and a calmodulin-like domain [9], [11], [12], [13]. The N-terminal domain is highly variable and contains myristoylation or palmotylation sites which may contribute to membrane localization [12], [14]. And the calmodulin-like domain contains EF-hands structure for binding of Ca^2+^ [11], [12].

It was confirmed that CDPK genes were involved in regulating plant response to various stimuli, including abiotic and biotic stresses and hormones. Overexpression of the rice CDPK gene *OsCDPK7* enhanced the tolerance of the transgenic rice plants to cold, salt, and drought stress [15], [16], and *OsCPK12* overexpression plants increased the tolerance to salt stress but to blast fungus [17]. The low temperatures induced *ZmCDPK1* expression in maize [18], and *ZmCPK11* participated in touch- and wound-induced pathways [19]. In tobacco, *NtCPK4* expression was increased in response to the treatment of gibberellin or NaCl [20]. In Arabidopsis, *AtCPK10* was reported to participate in ABA- and Ca^2+^-medicated stomatal regulation in response to drought stress [21], and *AtCPK3* and *AtCPK6* were also shown as positive transducers of stomatal ABA signaling in guard cells [22]. *AtCPK23* was demonstrated to function in response to drought and salt stresses [23]. In addition, some CDPK genes were also involved in pollen tube growth [24], root development [25], cell division and differentiation, and cell death [26], [27].

So far, many CDPK genes have been identified from numerous plant species. In Arabidopsis, 34 CDPK genes were revealed [9], [12]. And similarly, 31 CDPK genes in rice genome has identified by a genome-wide analysis [28]. In wheat, 20 CDPK genes including 14 full-length cDNA sequences were comprehensively studied [29]. And in poplar, 30 CDPK genes were identified [30], and 35 CDPK genes were initially revealed in maize genome [31]. Other higher plants such as tomato [32], potato [33], [34], tobacco [35], [36] and grapevine [37] also have multiple CDPK genes characterized in recent years. However, compared to the extensive studies of CDPK genes in many other plant species, little is known about this gene family in cotton. Till now, only one CDPK gene, *GhCPK1*, has been identified from upland cotton. And its transcripts accumulated primarily in the elongating fiber, which suggested that *GhCPK1* might play a vital role in the calcium signaling events associated with fiber elongation [38], [39].

Cotton is the major source of natural fibers used in the textile industry and is cultivated worldwide. Cotton, which belongs to the genus of *Gossypium*, includes approximately 45 diploid and five tetraploid species and serves as an excellent model system for evolutionary studies of polyploidy plants [40]. With completion of the genome sequencing of the diploid cotton *Gossypium raimondii* [41], genome-wide analysis of all the genes belonging to specific gene families have been realized. Here, 41 CDPK genes from *G. raimondii* were identified by database searches and classified according to phylogenetic analysis. Furthermore, the expression profiles of CDPK genes in response to low temperature, which affects seed germination,plant development, and final yield in cotton, were investigated. The identification and comprehensive study for CDPK genes from *G. raimondii* will provide valuable information for breeding stress-resistant cotton and further studying of the biological function and evolutionary relationship of this family in cotton.

## Materials and Methods

### Gene retrieval and annotation

The *G. raimondii* genome database (release v2.1) [41] was downloaded from Phytozome (http://www.phytozome.net/cotton.php). The published CDPK protein information for Arabidopsis [12] and rice [28] were obtained from the Arabidopsis Information Resource (TAIR release 10, http://www.arabidopsis.org) and the Rice Genome Annotation Project Database (RGAP release 7, http://rice.plantbiology.msu.edu/index.shtml), respectively. To identify potential CDPK genes in *G. raimondii*, the Arabidopsis and rice CDPK proteins were used as queries by searching against the *G. raimondii* genome database using BlastP and tBlastN programs with default parameters. Subsequently, all hits were verified with the InterProScan program (http://www.ebi.ac.uk/Tools/pfa/iprscan/) [42] to confirm the presence of the protein kinase domain. Finally, the Pfam (http://pfam.sanger.ac.uk/) [43] and SMART (http://smart.embl-heidelberg.de/) [44] database were applied to manually determine each candidate member of the CDPK family. The EF hand was predicted by ScanProsite tool (http://prosite.expasy.org/scanprosite/) [45]. The N-myristoylation motif and the palmitoylation site were predicted by Myristoylator (http://web.expasy.org/myristoylator/) [46] and CSS-Plam program [47], respectively. The molecular weight (MW) of the full-length protein was calculated by Compute pI/Mw tool (http://web.expasy.org/cgi-bin/compute_pi/pi_tool) [48].

### Phylogenetic analysis and Gene structure prediction

Multiple alignments of the full-length protein sequences were performed using Clustal X version 2.0 program [49] with default parameters. Phylogenetic trees were constructed using the method of Neighbor Joining MEGA 5.2 [50] with pairwise deletion option and poisson correction model. For statistical reliability, bootstrap tests were carried out with 1000 replicates.

The gene structures were obtained through comparing the genomic sequences and their predicted coding sequences of GSDS(http://gsds.cbi.pku.edu.cn/) [51].

### Chromosomal locations and gene duplications

All CDPK genes were mapped on the *G. raimondii* chromosomes according to their starting positions given in the genome annotation document. The chromosome location image was generated by MapInspect software [52].

Gene duplication events of CDPK genes in *G. raimondii* were also investigated. The gene duplication was defined according to (1) the length of aligned sequence cover >80% of the longer gene, (2) the identity of the aligned regions >80%, and (3) only one duplication event was counted for tightly-linked genes [53], [54]. With the chromosomal locations of CDPK genes, two types of gene duplications were recognized, i.e., tandem duplication and segmental duplication.

### Estimating Ka/Ks ratios for duplicated gene pairs

The CDPK duplicated gene pairs of *G.raimondii* were firstly aligned by Clustal X version 2.0 program [49]. Then Ks (synonymous substitution rate) and Ka (non-synonymous substitution rate) were calculated using the DnaSP v5.0 software (DNA polymorphism analysis) [55]. Finally, the Ka/Ks ratio was analyzed to assess the selection pressure for each gene pair.

### Cis-element analysis

For promoter analysis, 2000 bp genomic DNA sequences upstream of the initiation codon (ATG) were retrieved from the genome sequence. These sequences were subjected to search in the PLACE database (http://www.dna.affrc.go.jp/PLACE/signalscan.html) [56] to identify putative *cis*-elements in promoter regions.

### Plant materials and low temperature stress treatment

All the plants of *G. raimondii* were grown in a temperature-controlled chamber at 28°C with a photoperiod of 16 hours light and 8 hours dark. After ten days, the leaves, stems, roots, and cotyledons of some seedlings were collected to analyze tissue-specific expression, and the rest seedlings were used to examine the expression patterns of CDPK genes under low-temperature stress. Plant leaves of the seedlings grown in the temperature-controlled chamber treated at 10°C were harvested at 0, 3, 6, and 12 hours, which represented normal plants, slight stress, moderate stress, and severe stress, respectively. All collected samples were immediately frozen in liquid nitrogen and stored at −80°C.

### RNA isolation and quantitative real-time PCR (qRT-PCR)

Total RNA was extracted from all samples using EASYspin Plus RNAprep Kit, and the first-strand cDNAs were synthesized with PrimerScript 1st Strand cDNA synthesis kit (TaKaRa). All protocols followed the manufacturer's protocol. For quantitative real-time PCR (qRT-PCR) assay, gene-specific primers were designed for the 11 selected CDPK genes according to their CDSs (Table S1). The qRT-PCR was performed with SYBR premix Ex taq (TakaRa) and CFX96 Realtime System (BioRad) by strictly following the manufacturer's instructions. Each reaction was done in a final volume of 20 µl containing 10 µl of SYBR premix Ex taq, 1.0 µl of cDNA sample, and 0.5 µl of each gene-specific primer. The qRT-PCR cycles were conducted with 40 cycles and an annealing temperature of 60°C, the amplification programs were as follows: 95°C for 30 seconds, 40 cycles of 94°C for 10 seconds, 60°C for 10 seconds, and 72°C for 15 seconds. The cotton *UBQ7* gene was used as internal reference for all the qRT–PCR analyses. Each cDNA sample was tested in three replicates. The relative expression levels were calculated according to the 2^−△△Ct^ method [57]. The expression profiles were clustered using the Cluster 3.0 software [58].

## Results and Discussion

### Identification and annotation of CDPK genes in *G. raimondii*

The availability of the *G. raimondii* genome sequences [41] makes it possible to identify all CDPK gene family members in the diploid cotton. BLASTP and TBLASTN programs were performed to search the candidate CDPK genes from the *G. raimondii* genome with the query sequences of Arabidopsis and rice CDPK genes. Then Pfam and SMART analyses were used to verify the retrieved sequences, and finally a total of 41 non-redundant CDPK genes were confirmed and described (Table1). According to the proposed nomenclature for CDPK genes [59], we designated these 41 CDPK genes as *GrCPKs*, from *GrCPK1* to *GrCPK41*. The numbering of these *GrCPKs* was based on their position from top to bottom on corresponding chromosomes, from chromosome 1 to chromosome 13. Through analyzing the transcriptome sequencing data downloaded from the NCBI Sequence Read Archive (SRA), it was found that at least 37 *GrCPKs* were actively expressed in *G. raimondii* (Figure S1). The total number of CDPK genes identified from *G. raimondii* was greater than that in Arabidopsis [12] and rice [28]. The length and molecular weights (MW) of 41 GrCPK proteins were deduced from their predicted protein sequences. All identified GrCPK genes encoded proteins with amino acid numbers varying from 487 to 873 and molecular weight range between 54.6 kDa and 97.1 kDa, which were comparable with CDPK genes from other plant species [12], [28], [30], [31].

**Table 1:** The information of 41 CDPK genes from *G. raimondii*.

Gene symbol	ID	Chromosome	Alternative splicing	CDS	Amino acid	MW (kDa)	No. of EF hands	N-terminal aa	N-Myristoylation	N-palmitoylation
GrCPK1	Gorai.001G135000	1	_	1542	513	57.3	4	MGNCCSRG	Yes	Yes
GrCPK2	Gorai.001G138000	1	2	1590	529	59.5	2	MGNCCATT	No	Yes
GrCPK3	Gorai.002G088800	2	4	1614	537	60.5	4	MGSCLTKS	Yes	Yes
GrCPK4	Gorai.002G153600	2	2	1776	591	66.3	4	MGNSCAKS	Yes	Yes
GrCPK5	Gorai.003G009500	3	8	1626	541	61.3	4	MGACLSAT	Yes	Yes
GrCPK6	Gorai.003G084000	3	_	1527	508	57.5	3	MGNCNGLP	No	Yes
GrCPK7	Gorai.003G092900	3	3	1608	535	60.6	3	MGNCCATP	Yes	Yes
GrCPK8	Gorai.004G015100	4	_	1617	538	60.5	4	MGNCCSRG	Yes	Yes
GrCPK9	Gorai.005G019800	5	_	1599	532	60.7	4	MGSCVARP	Yes	Yes
GrCPK10	Gorai.005G074300	5	_	1527	508	56.9	4	MNNQSSSI	No	No
GrCPK11	Gorai.005G216500	5	6	1707	568	63.6	4	MGNTCRGS	No	Yes
GrCPK12	Gorai.006G124800	6	_	1575	524	58.6	4	MGNCCSCG	Yes	Yes
GrCPK13	Gorai.006G128200	6	3	1596	531	59.8	3	MGNCCATP	No	Yes
GrCPK14	Gorai.006G137800	6	_	1596	531	60.2	3	MGNCCVTS	No	Yes
GrCPK15	Gorai.006G147600	6	_	1836	611	68.4	4	MGNNCFKT	No	Yes
GrCPK16	Gorai.007G025000	7	5	1593	530	60.4	4	MGNCNRPP	No	Yes
GrCPK17	Gorai.007G035100	7	3	1653	550	62.3	4	MGNCNACV	No	Yes
GrCPK18	Gorai.007G194500	7	3	1665	554	62.8	4	MGACLSTT	Yes	Yes
GrCPK19	Gorai.007G378700	7	_	1584	527	59.2	3	MGNCCRSP	No	Yes
GrCPK20	Gorai.008G013700	8	_	1728	575	64.6	4	MRLHYCMR	No	Yes
GrCPK21	Gorai.008G251000	8	2	1605	534	60.2	4	MGNCNSQP	Yes	Yes
GrCPK22	Gorai.009G078000	9	_	1599	532	59.5	4	MGNLCSRS	Yes	Yes
GrCPK23	Gorai.009G191500	9	_	2622	873	97.1	4	MGICQSLC	No	Yes
GrCPK24	Gorai.009G290200	9	5	1617	538	60.4	4	MNKKIAGS	No	No
GrCPK25	Gorai.009G351200	9	_	1605	534	60.7	4	MGSCISAP	Yes	Yes
GrCPK26	Gorai.009G394700	9	_	1947	648	71.8	4	MGNVCATL	No	Yes
GrCPK27	Gorai.009G395400	9	5	1719	572	63.6	4	MGNACAGP	No	Yes
GrCPK28	Gorai.009G438300	9	_	1575	524	58.7	4	MGGCLTKT	Yes	Yes
GrCPK29	Gorai.010G001300	10	_	1581	526	59.1	4	MGLCQSLG	Yes	Yes
GrCPK30	Gorai.010G252400	10	_	1584	527	59.3	4	MGCCSSKN	Yes	Yes
GrCPK31	Gorai.011G014200	11	_	1656	551	62.0	4	MGCFSSKH	Yes	Yes
GrCPK32	Gorai.011G098300	11	_	1635	544	62.0	4	MGICLSTT	Yes	Yes
GrCPK33	Gorai.011G228500	11	5	1764	587	65.4	4	MGNTCVGP	No	Yes
GrCPK34	Gorai.012G045700	12	2	1494	497	55.9	4	MSRTSSGT	No	No
GrCPK35	Gorai.012G114600	12	_	1584	527	59.4	3	MGNCCRSP	No	Yes
GrCPK36	Gorai.012G138900	12	2	1659	552	62.0	4	MGNTCRGP	No	Yes
GrCPK37	Gorai.013G003100	13	2	1740	579	64.6	4	MGNTCVGP	No	Yes
GrCPK38	Gorai.013G064400	13	5	1464	487	54.6	4	MRRAIDHQ	No	No
GrCPK39	Gorai.013G064500	13	2	1572	523	58.3	3	MGNTCLGS	No	Yes
GrCPK40	Gorai.013G159300	13	3	1611	536	60.4	4	MGGCLTKN	Yes	Yes
GrCPK41	Gorai.013G253100	13	_	1584	527	58.8	4	MGNCCTRG	No	Yes

_ represents no alternative splice variants.

All 41 CDPKs identified in our study possessed the typical CDPK structure with four characterized domains, including a variable N-terminal domain, a catalytic Ser/Thr protein kinase domain, an autoinhibitory region, and a calmodulin-like domain. The N-terminal domain of several CDPK proteins contained myristoylation motif with a Gly residue at the second position, which was thought to be critical for mediating protein-protein and protein-membrane interactions [60]. It was reported that CDPK genes which had an N-myristoylation motif tended to localize in the plasma membranes [14], [21], [29], [61], [62], demonstrating that myristoylation was required for membrane binding. In addition, palmitoylation, as a second type of lipid modification, was also necessary to stabilize for membrane association [14]. Among 41 CDPKs in our study, 18 CDPKs were predicted to have myristoylation motifs at the N-terminus, and all of them possessed at least one palmitoylation site. However, the membrane association of CDPKs was complex and might be affected by other motifs. For example, *TaCPK3* and *TaCPK15* lacking myristoylation motifs could also be associated with the membranes [29], while *ZmCPK1* which was predicted to have an N-myristoylation motif was found to localize into the cytoplasm and nucleus [63]. Therefore, the subcellular localizations of these CDPKs were still needed to be further characterized experimentally.

The calmodulin-like domain contained Ca^2+^ binding EF hand structure that allow CDPK proteins to function as a Ca^2+^ sensor. In *G. raimondii*, 33 CDPKs contained four Ca^2+^ binding EF hands, seven CDPKs, including GrCPK6, GrCPK7, GrCPK13, GrCPK14, GrCPK19, GrCPK35, and GrCPK39, had three EF hand motifs each, whereas GrCPK2 had two EF hand motifs only. This difference in EF hand was also found in the CDPK family of other plants [9], [30], [31], [64]. Studies have demonstrated that the number and position of EF hands might be important for determining the Ca^2+^ regulation of CDPK activity [65], [66], and compared to C-terminal EF3 and EF4 motifs, N-terminal EF1 and EF2 motifs with lower Ca^2+^-binding affinities were more important for activating the kinases [67], [68].

### Phylogenetic analysis of the CDPK gene family

**Figure 1:**
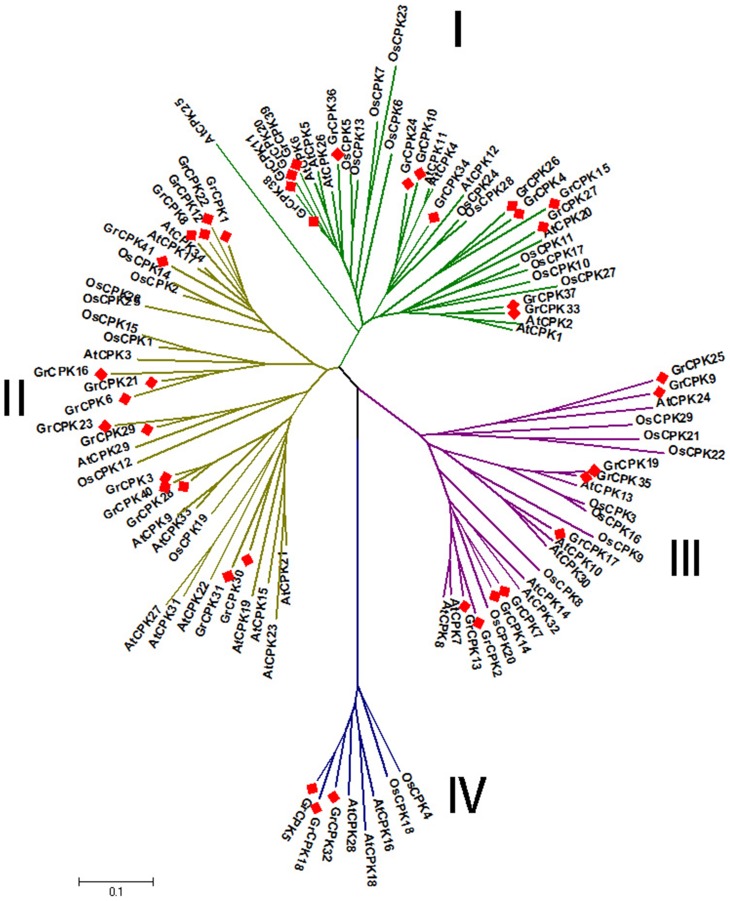
Unrooted phylogenetic tree of CDPK genes from *G. raimondii*, Arabidopsis and rice. Four subfamilies are labeled as I, II, III, and IV. And the branches of each subfamily are indicated in a specific color.

To detect the evolutionary relationships of CDPK genes, an unrooted phylogenetic tree was generated from alignments of the full-length sequences of *G. raimondii*, Arabidopsis and rice CDPK proteins. All the CDPK proteins were divided into four subgroups (Figure 1): CDPK I, CDPK II, CDPK III and CDPK IV. CDPK I contained 14 CDPKs from *G. raimondii*, 11 from rice, and ten from Arabidopsis. CDPK II contained 15 CDPKs from *G. raimondii*, eight from rice, and 13 from Arabidopsis. The CDPKs numbers in CDPK I and CDPK II from *G. raimondii* were greater than that from Arabidopsis and rice, mainly due to more CDPK genes in *G. raimondii*. In CDPK III, there were nine CDPKs from *G. raimondii*, eight from rice, and eight from Arabidopsis, while CDPK IV genes consisted of the smallest subfamilies in all of the three species, which contained three CDPKs from *G. raimondii*, two from rice, and three from Arabidopsis. And the number of genes in CDPK III and CDPK IV were approximately identical across these three species. Interestingly, among the four subfamilies, CDPK I in rice comprised the most members, while CDPK II in *G. raimondii* and Arabidopsis was the largest subfamily. Recent study indicated that poplar had total 30 CDPK genes with 11 genes in CDPK I, eight genes in CDPK II, nine genes in CDPK III and two genes in CDPK IV [30]. These results implied that the difference in the number of CDPK genes was mainly due to the occurrence of gene gain or loss in CDPK I or CDPK II subfamilies independently among the different organisms.

Phylogenetic analysis also showed that *GrCPKs* were more closely allied to *AtCPKs* than to *OsCPKs*, consistent with the evolutionary relationships among *G. raimondii*, Arabidopsis, and rice. Moreover, all the CDPK genes from the three species sorted into four distinct clades, implying that these four subfamilies existed before the divergence of monocots and dicots, which also supported the hypothesis that CDPK genes radiated into four subfamilies before algae and land plants split [69].

### Structural analysis and chromosomal localization of GrCPKs

**Figure 2:**
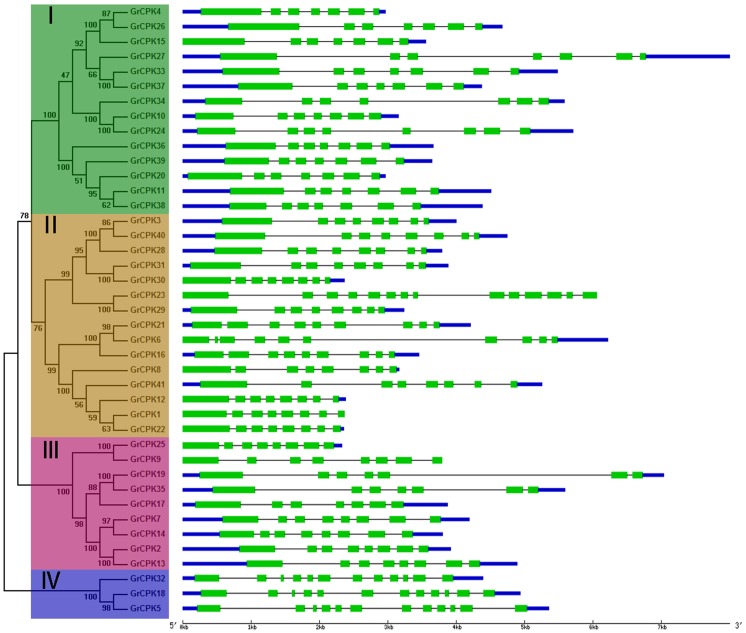
Phylogenetic relationship and gene structure of CDPK genes from *G. raimondii*. Four subfamilies labeled as I, II, III, and IV are marked with different color backgrounds. Exons are represented by green boxes and introns by black lines. The untranslated regions (UTRs) are indicated by thick blue lines.

To get further insight into the possible structural evolution of *GrCPKs*, a separate unrooted phylogenetic tree was constructed using the protein sequences of all the CDPK genes from *G. raimondii*, and then the diverse exon-intron organizations of *GrCPKs* were compared. As shown in Figure 2, the *GrCPKs* clustered in the same subfamily shared very similar exon-intron structures. Most members in Group I possessed seven exons, except for *GrCPK24*, which contained eight exons. Most genes in Group II had eight exons, but *GrCPK16* and *GrCPK21* had nine exons each, *GrCPK6* contained ten exons, and *GrCPK23* contained 14 exons. Six genes in group III had eight exons, whereas three genes contained seven exons each. All genes in Group IV contained 12 exons. These conserved gene structure within each group supported their close evolutionary relationship.

**Figure 3:**
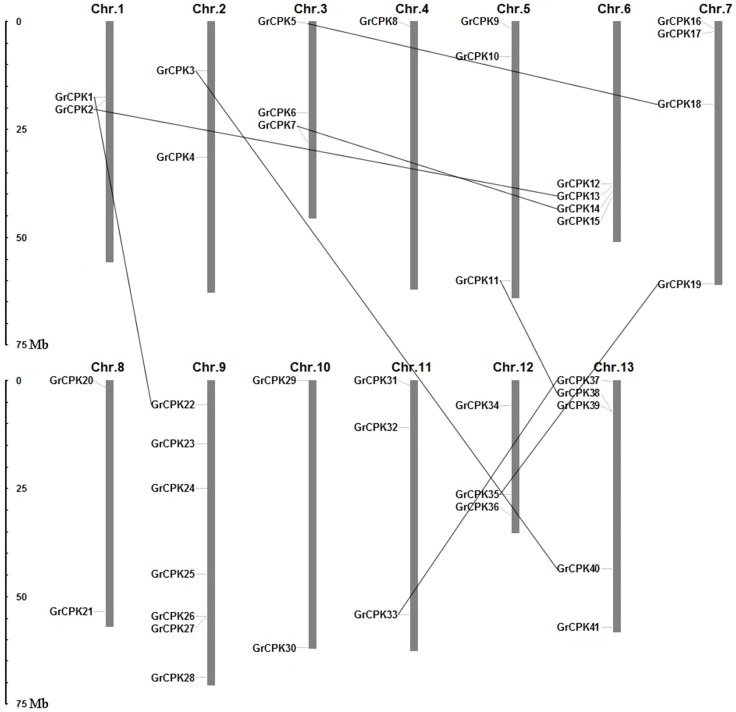
Distributions of CDPK genes from *G. raimondii* in 13 chromosomes. Chromosome numbers are indicated above each vertical bar. The scale represents megabases (Mb). The duplicated gene pairs are connected with black lines.

The 41 nonredundant CDPK genes were mapped on the 13 *G. raimondii* chromosomes (Figure 3). They were found to be distributed unevenly among 13 chromosomes and the number of CDPK genes on each chromosome varied widely. Chromosome 9 which contained 7 CDPK genes was the highest one in gene numbers, followed by chromosome 13 on which five genes were found. Chromosome 6 and 7 both had four genes, and chromosome 3, 5, 11, and 12 contained three genes each. Chromosome 1, 2, 8, and 10 had two genes each only, whereas only single CDPK gene was localized on chromosome 4.

### CDPK gene duplications and functional divergence

To elucidate the expanded mechanism of the CDPK gene family in *G. raimondii*, gene duplication events, including tandem and segmental duplications, were investigated, which were thought to play a significant role in the amplification of gene family members in the genome [70], [71]. A total of eight duplication events, *GrCPK2*/*GrCPK13*, *GrCPK7*/*GrCPK14*, *GrCPK18*/*GrCPK5*, *GrCPK19*/*GrCPK35*, *GrCPK22*/*GrCPK1*, *GrCPK37*/*GrCPK33*, *GrCPK38*/*GrCPK11*, and *GrCPK40*/*GrCPK3*, were found in the *G. raimondii* genome and all of them were segmental duplication events based on the chromosomal distribution of the CDPK genes (Figure 3). This result suggested that the CDPK gene family expansion in *G. raimondii* was mainly attributed to segmental duplication events.

The duplicated gene pairs might experience three alternative outcomes during the process of evolution, i.e., (i) one copy may simply become silenced and lost original functions (nonfunctionalization); (ii) one copy may acquire a novel, beneficial function, with the other copy retaining the original function (neofunctionalization); or (iii) both copies may become partition of original functions (subfunctionalization) [72]. We subsequently calculated the non-synonymous to synonymous substitution ratio (Ka/Ks) for each pair of duplicated CDPK genes, which showed the selective force acting on the protein, to reveal whether Darwinian positive selection was associated with functional divergence after gene duplications. Generally, Ka/Ks >1 indicates positive selection, Ka/Ks  =  1 indicates neutral selection, while Ka/Ks <1 indicates negative or purifying selection. In this study, the Ka/Ks ratios for eight duplicated CDPK gene pairs were no larger than 0.2 (Table 2), which demonstrated that the CDPK genes from *G. raimondii* had mainly experienced strong purifying selection pressure with limited functional divergence after segmental duplications. These results suggested that functions of the duplicated CDPK genes did not diverge much during subsequent evolution.

**Table 2:** Ka/Ks analysis for the duplicated gene pairs.

Duplicated gene 1	Duplicated gene 2	Ka	Ks	Ka/Ks	Purifying selection	Duplicate type
*GrCPK2*	*GrCPK13*	0.068	0.422	0.161	Yes	Segmental
*GrCPK14*	*GrCPK7*	0.083	0.484	0.171	Yes	Segmental
*GrCPK18*	*GrCPK5*	0.057	0.589	0.096	Yes	Segmental
*GrCPK19*	*GrCPK35*	0.028	0.399	0.071	Yes	Segmental
*GrCPK22*	*GrCPK1*	0.080	0.683	0.118	Yes	Segmental
*GrCPK37*	*GrCPK33*	0.062	0.511	0.121	Yes	Segmental
*GrCPK38*	*GrCPK11*	0.034	0.515	0.065	Yes	Segmental
*GrCPK40*	*GrCPK3*	0.038	0.528	0.071	Yes	Segmental

### Expression analysis of the CDPK genes in *G. raimondii*

Cold stress is one of the serious environmental stresses that most land plants might encounter during the process of their growth. And so far, numerous of CDPK genes identified in various plant species have been proven to play crucial roles in cold stress response. Cotton is a subtropical crop and its cultivation has been extended from tropical and subtropical to colder regions. Low temperature (<15°C ) can adversely affect plant development, resulting in poor germination, infection by fungi and other disease-causing organisms, and yield loss [73]. There is little information about the functions of CDPK genes in the cotton response to low temperature.

The *cis*-element analysis in promoter regions might provide some indirect evidence for the functional dissection of CDPK genes in stress responses [74]. To investigate the potential functions of *GrCPKs* in cotton under cold stress, the possible presence of low temperature-responsive element (LTRE) in the promoter regions of all the *GrCPKs* was detected by searching against the PLACE database (Table S2). The results revealed that the majority, 27 out of 41, of *GrCPKs* contained the LTRE in promoter sequences, indicating that these *GrCPKs* might participate in the signal transduction of the plant response to cold stress. In order to verify the expression patterns of these genes, eight *GrCPKs* which had the LTRE in promoter regions were selected for qRT-PCR analysis. In addition, as the gene expression was a complex process, the existence of some *cis*-elements did not always correlate with the gene function [74], therefore, three *GrCPKs*, *GrCPK2*, *GrCPK11*, and *GrCPK27*, which had no LTRE in promoter regions were also selected in this expression analysis.

**Figure 4:**
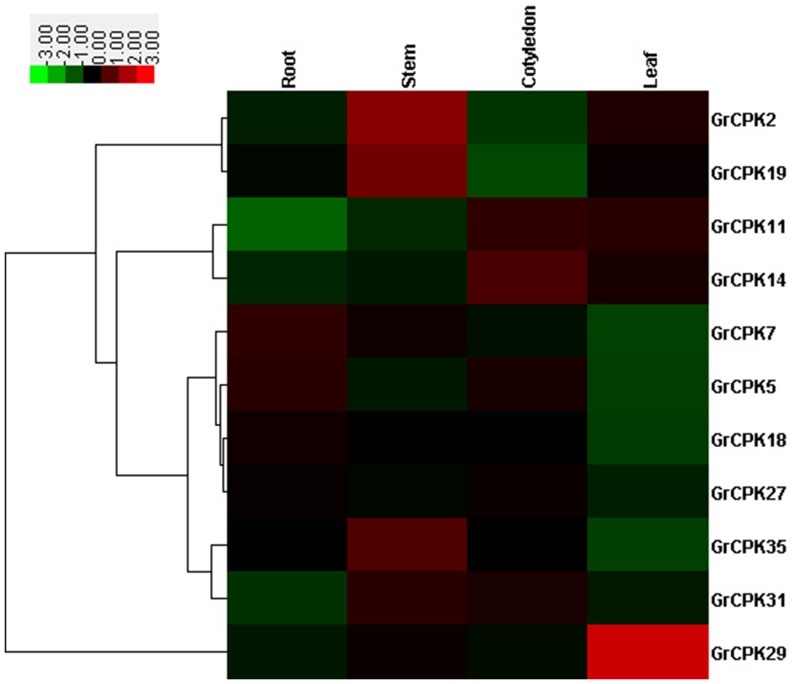
Expression patterns of 11 *GrCPKs* in four representative tissues of *G. raimondii* seedlings. The color bar represents the relative signal intensity values.

Firstly, qRT-PCR analysis was performed to examine expression patterns of 11 selected *GrCPKs* in roots, stems, cotyledons, and leaves of 10-day-old seedlings. As shown in Figure 4, *GrCPK5* and *GrCPK7* were expressed at high levels in roots, while *GrCPK2*, *GrCPK19*, and *GrCPK35* shared high expressions in young stems. In cotyledons, *GrCPK14* exhibited relatively high transcript abundance, whereas *GrCPK29* were predominantly expressed in leaves, which showed about 6-fold higher expression than all other tissues. The results demonstrated that most of these 11 *GrCPKs* had specific spatial expression patterns, which was similar with CDPK genes in maize [54] and rice [74] that also exhibited tissue-specific expression.

**Figure 5:**
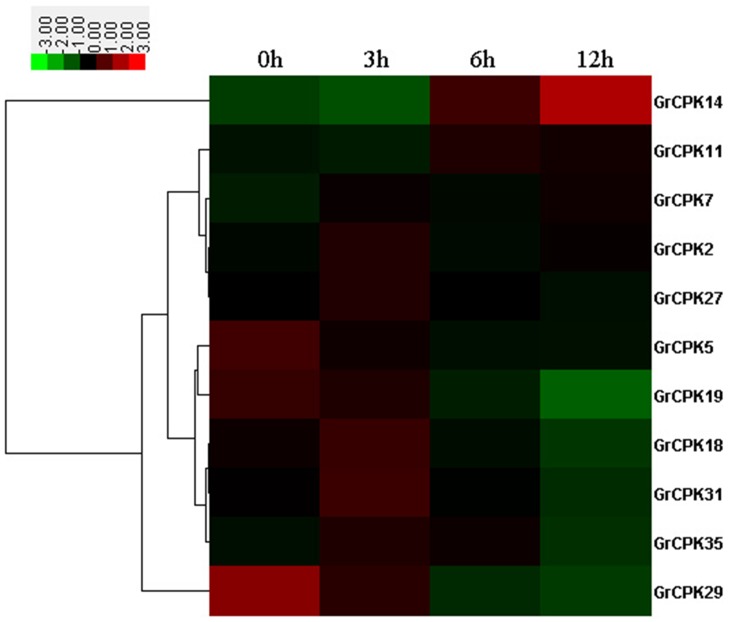
Expression profiling of 11 *GrCPKs* in leaves under 10°C treatment. The color bar represents the relative signal intensity values.

To further confirm the cold stress response of *GrCPKs*, the expression patterns of 11 *GrCPKs* in 10-day-old leaves under low temperature stress of 10°C were investigated. The expression levels of all the 11 CDPK genes responsive to slight cold stress (3 hours), moderate cold stress (6 hours), and severe cold stress (12 hours) were shown in Figure 5, comparing with normal plants. The result showed that the expression of all the 11 *GrCPKs* were induced or repressed by clod treatment. Among them, *GrCPK2*, *GrCPK7*, *GrCPK11*, *GrCPK14*, *GrCPK18*, *GrCPK27*, *GrCPK31*, and *GrCPK35* were positively regulated response to cold stress according to their transcription levels. For example, *GrCPK14* showed a significantly increase in its expression after long time cold stress treatment. Whereas, the transcription levels of the other three genes, *GrCPK5*, *GrCPK19*, and *GrCPK29*, were down-regulated in response to cold stress.

## Conclusions

A complete set of 41 CDPK genes were identified in the *G. raimondii* genome, which were categorized into four subgroups and mapped on the 13 chromosomes. Segmental duplication contributed to the expansion of *GrCPKs*, and the duplicated genes mainly undergone strong purifying selection pressure with limited functional divergence. The majority of *GrCPKs* contained the low temperature-responsive elements in their promoter sequences. The expression of *GrCPKs* were also induced or repressed by the clod stress treatment. The results would be helpful for better understanding of the biological functions of the CDPK genes in cotton and the evolutionary relationship of this family in *Gossypium* species.

## Supporting Information

Figure S1 **Expression analysis of** ***GrCPKs*** **using the transcriptome sequencing data.** The transcriptome sequencing datasets of *G. raimondii* for three tissue samples (mature leaves, 0DPA ovules, and 3DPA ovules) were downloaded from the NCBI Sequence Read Archive (SRA) with accession numbers SRX111367, SRX111365 and SRX111366. Then sequenced reads of these three datasets were mapped to the sequences of *GrCPKs*, respectively. And matches were converted to RPKM to estimate gene expression levels. The expression profiles were clustered using the Cluster 3.0 software. The color bar represents the relative signal intensity values. DPA: Days Post Anthesis.
(TIF)

Table S1 **PCR primers used in this study.**
(XLSX)

Table S2 **The LTRE in the promoter regions of** ***GrCPKs*** **.**
(XLSX)
