# Supplementary figures and images for: Genome-Wide Survey and Expression Analysis of Calcium-Dependent Protein Kinase in Gossypium raimondii
Source: PLoS One. 2014 Jun 2;9(6):e98189. doi: 10.1371/journal.pone.0098189 (PMC4041719; doi:10.1371/journal.pone.0098189)

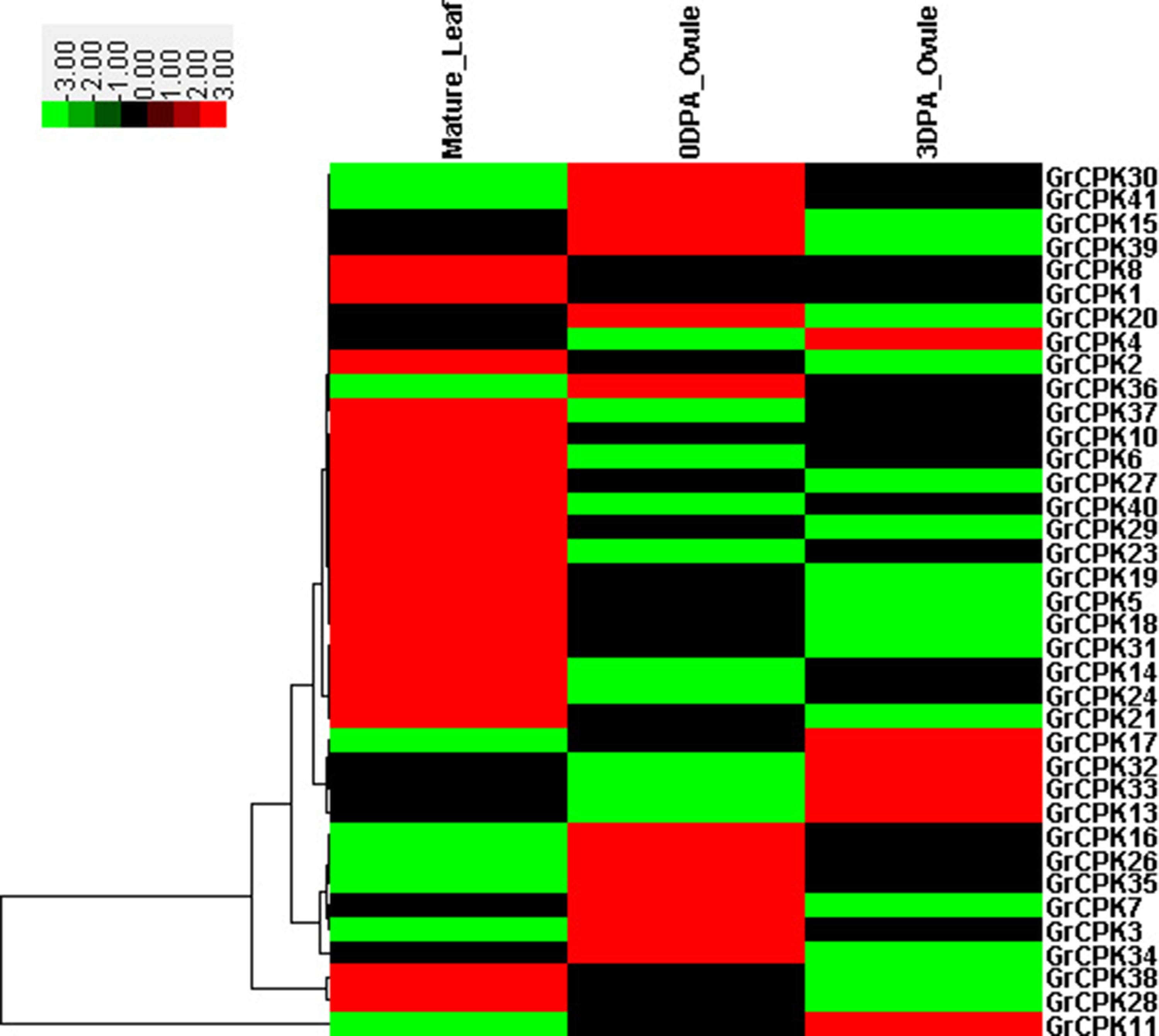

Supplement: Figure S1 — Expression analysis of GrCPKs using the transcriptome sequencing data. The transcriptome sequencing datasets of G. raimondii for three tissue samples (mature leaves, 0DPA ovules, and 3DPA ovules) were downloaded from the NCBI Sequence Read Archive (SRA) with accession numbers SRX111367, SRX111365 and SRX111366. Then sequenced reads of these three datasets were mapped to the sequences of GrCPKs, respectively. And matches were converted to RPKM to estimate gene expression levels. The expression profiles were clustered using the Cluster 3.0 software. The color bar represents the relative signal intensity values. DPA: Days Post Anthesis. (TIF) [file pone.0098189.s001.tif]
